# Graphene/PVA buckypaper for strain sensing application

**DOI:** 10.1038/s41598-020-77139-2

**Published:** 2020-11-18

**Authors:** Ahsan Mehmood, N. M. Mubarak, Mohammad Khalid, Priyanka Jagadish, Rashmi Walvekar, E. C. Abdullah

**Affiliations:** 1 Department of Chemical Engineering, Faculty of Engineering and Science, Curtin University, 98009 Kuching, Sarawak Malaysia; 2 Graphene & Advanced 2D Materials Research Group (GAMRG), School of Science and Technology, Sunway University, No. 5, Jalan Universiti, Bandar Sunway, 47500 Subang Jaya, Selangor Malaysia; 3 School of Energy and Chemical Engineering, Department of Chemical Engineering, Xiamen University Malaysia, Jalan Sunsuria, Bandar Sunsuria, 43900 Sepang, Selangor Malaysia; 4 Department of Chemical Process Engineering, Malaysia-Japan International Institute of Technology (MJIIT), Universiti Teknologi Malaysia (UTM), Jalan Sultan Yahya Petra, 54100 Kuala Lumpur, Malaysia

**Keywords:** Health care, Energy science and technology, Materials science, Nanoscience and technology

## Abstract

Strain sensors in the form of buckypaper (BP) infiltrated with various polymers are considered a viable option for strain sensor applications such as structural health monitoring and human motion detection. Graphene has outstanding properties in terms of strength, heat and current conduction, optics, and many more. However, graphene in the form of BP has not been considered earlier for strain sensing applications. In this work, graphene-based BP infiltrated with polyvinyl alcohol (PVA) was synthesized by vacuum filtration technique and polymer intercalation. First, Graphene oxide (GO) was prepared via treatment with sulphuric acid and nitric acid. Whereas, to obtain high-quality BP, GO was sonicated in ethanol for 20 min with sonication intensity of 60%. FTIR studies confirmed the oxygenated groups on the surface of GO while the dispersion characteristics were validated using zeta potential analysis. The nanocomposite was synthesized by varying BP and PVA concentrations. Mechanical and electrical properties were measured using a computerized tensile testing machine, two probe method, and hall effect, respectively. The electrical conducting properties of the nanocomposites decreased with increasing PVA content; likewise, electron mobility also decreased while electrical resistance increased. The optimization study reports the highest mechanical properties such as tensile strength, Young’s Modulus, and elongation at break of 200.55 MPa, 6.59 GPa, and 6.79%, respectively. Finally, electrochemical testing in a strain range of ε ~ 4% also testifies superior strain sensing properties of 60 wt% graphene BP/PVA with a demonstration of repeatability, accuracy, and preciseness for five loading and unloading cycles with a gauge factor of 1.33. Thus, results prove the usefulness of the nanocomposite for commercial and industrial applications.

## Introduction

Flexible strain sensors, in contrast to metal or semiconductor-based conventional strain sensors, exhibit a superior application potential in a wide array of fields that includes monitoring of sports performance^1^, virtual reality^2^, personal healthcare^3^, human–machine interface^4^, and so on. Their ability to transform mechanical deformation stimuli into electrical shift signals is largely appreciated by researchers as well as industrial guys^5^. At large, flexible sensors are principally divided into four categories, namely resistance type, capacitance type, triboelectric, and piezoresistive type^6^. Henceforth, the piezoresistive type is usually synthesized by a facile method, with low power consumption, convenient signal acquisition, and a simple readout mechanism^7^. Thus, various materials have been tried to obtain a highly efficient, flexible, sensitive, and stretchable strain sensor. However, nanomaterials are highly regarded for their abilities to satisfy all these criteria and provide further room for research and development.

Graphene is such material that proves to be a promising candidate as the flexible sensor, on account of its outstanding flexibility, high electrical conductivity, chemical stability, and lightweight^8^. Most significantly, the tremendous attention it gathered among technological and scientific communities due to superior mechanical and electrical properties and high frequency which go up to THz^9^. Graphene is a material of immense strength with young’s modulus of 1100 Gpa, the fracture strength of 125 GPa, specific surface area of 2630 m^2^/g, the thermal conductivity of 5000 W/mK, the thickness of 0.345 nm, excellent transport phenomena, zero bandgaps, and mobility charge carrier of 250,000 cm^2^/V^2^s^10^. Among other extraordinary features of graphene, strong π-π stacking, and forces of attraction such as van der Waals among planar basal planes of graphene layers that allow them to aggregate, which results in an ability to develop a controllable self-assembled structure^11^. These extraordinary properties of graphene endow graphene with novel technological opportunities, in the form of promising nanofiller material for the sole purpose of reinforcement of composites, in contrast to other carbon nanomaterials. Thus graphene stands out among other materials and ideal for developing 3-dimensional structures^12^. Furthermore, the defects of carbon nanotubes in orientation and geometry can be compensated by graphene sheets. This unique characteristic of graphene has profound implications and thus gives the lead to the era of ultra-sensitive strain sensors. Thus graphene is described as a “miracle material” has demonstrated its sheer potential for the synthesis of graphene-based strain sensors^10^.

In the field of polymer nanocomposites, graphene proves itself to be a promising modifier due to its astonishing properties. However, the whole effort on polymer nanocomposite is in line to modify neat polymer to attain new and better properties without adding excessive weight or sacrificing processability^13^. However, advancements have been made with the use of graphene as a modifier in polymer matrices, whereas no considerable success has been achieved due to poor compatibility between polymer matrix and pristine graphene. Though, as compared to pristine graphene, abundantly present oxygen functional group in graphene oxide not only allows better compatibility and dispersion in aqueous solution but also provides a strong connection between GO and polymer matrix through non-covalent and covalent bonds^14^. Thus, that makes GO a better choice to be used to prepare graphene oxide/polymer nanocomposite in the form of buckypaper (BP) and study its strain sensing properties.

Buckypaper is a thin porous freestanding network of carbon fillers bounded by van der Waals forces of interaction and in appearance, it looks like paper-like material as its name indicates. It is fabricated through various techniques, namely the spin coating technique, evaporation induced, vacuum filtration method, self-assembly technique, and resin infiltration technique^15^. However, polymeric based buckypaper has achieved much success and has been able to seek superior attention to vast sensing applications. The graphene sheets have interlayer interaction due to van der Wall forces of attraction that are relatively weak and have slippage as a result of sliding between layers of graphene. Thus, these issues have the potential to disturb the strain sensing properties of strain sensors^16^. The buckypaper infiltrated with polymer matrix is viewed as one with better strain transfer phenomena across the composite by providing better binding interfacial bonding. The polymer infiltration into the porous graphene network improves interfacial adhesion among graphene and polymer, which results in a strong bond between graphene and polymer and forms a helical polymer structure. The current method of synthesis has provided a solution to the intrinsic tendency of graphene sheets to agglomerate due to van der Waals forces of attraction, the high viscosity of graphene/polymer suspension, and low solubility in solvents. Whereas, the graphene oxide produced initially had essential functional groups to make strong bonds with polyvinyl alcohol and produced a buckypaper stain sensor with strong interfacial bonding among graphene sheets and polymer matrix^17^. Han, et al.^18^ synthesized Carbon nanotube-based buckypaper infiltrated with a polymer matrix of epoxy resin that helped to achieve significant mechanical properties such as tensile strength, Young’s modulus, and elongation-at-break of 146.1 Mpa, 13.8 GPa, and 1.3% respectively. Whereas, Yee et al.^17^ synthesized multiwalled carbon nanotube-based buckypaper infiltrated polyvinyl alcohol with superior mechanical properties such as tensile strength, Young’s modulus, and elongation-at-break of 156.28 MPa, 4.02 GPa, and 5.85%, respectively. The strain sensor synthesized in this work demonstrated better mechanical properties such as tensile strength, Young’s modulus, and elongation-at-break of 200.55 MPa, 6.59 GPa, and 6.79%, respectively. Another important aspect of the strain sensor is its gauge factor which the selected sample has of 1.33, which is higher than reported gauge factors of buckypaper strain sensors such as 0.49^19^, 0.34^20^, and 0.9 to 1.3^21^. Hence, this strain sensor proves to be a significant addition to the existing range of strain sensors and may provide a wide range of applications in various fields of life.

In this work, a graphene-based strain sensor infiltrated with polyvinyl alcohol (PVA) is reported. The buckypaper is usually synthesized from carbon nanotubes, but here we report a graphene-based buckypaper, which is the main novelty of the work. The other process of synthesis follows a facile technique, which is reproducible and requires limited equipment for the synthesis. Whereas, the nanocomposite material has superior mechanical properties in terms of tensile strength, young’s modulus, and elongation at break as compared to other buckypaper strain sensors. Graphene as a material has promising characteristics, in terms of superior mechanical and electrical properties, flexibility, and high thermal conductivity. Thus, graphene exhibited a bright prospect for strain sensor application. A nanocomposite material consisting of graphene oxide and polyvinyl alcohol was prepared by varying concentrations of 45, 60, and 75 wt% through simple vacuum filtration and polymer intercalation. The motivation behind the use of PVA for infiltration is supported by past studies demonstrated by several researchers^17,22,23^. Regardless, polyvinyl alcohol has high applicability for strain sensing applications, is biodegradable, synthesized artificially, and readily available commercially^24^. Samples were evaluated through mechanical, electrical, electromechanical, and morphological techniques to determine the sample properties. The research findings were consistent with the identification of optimal conditions and concentration ratio of PVA and graphene to synthesize PVA infiltrated BP for strain sensing.

## Characterization studies

### FTIR analysis

**Figure 1:**
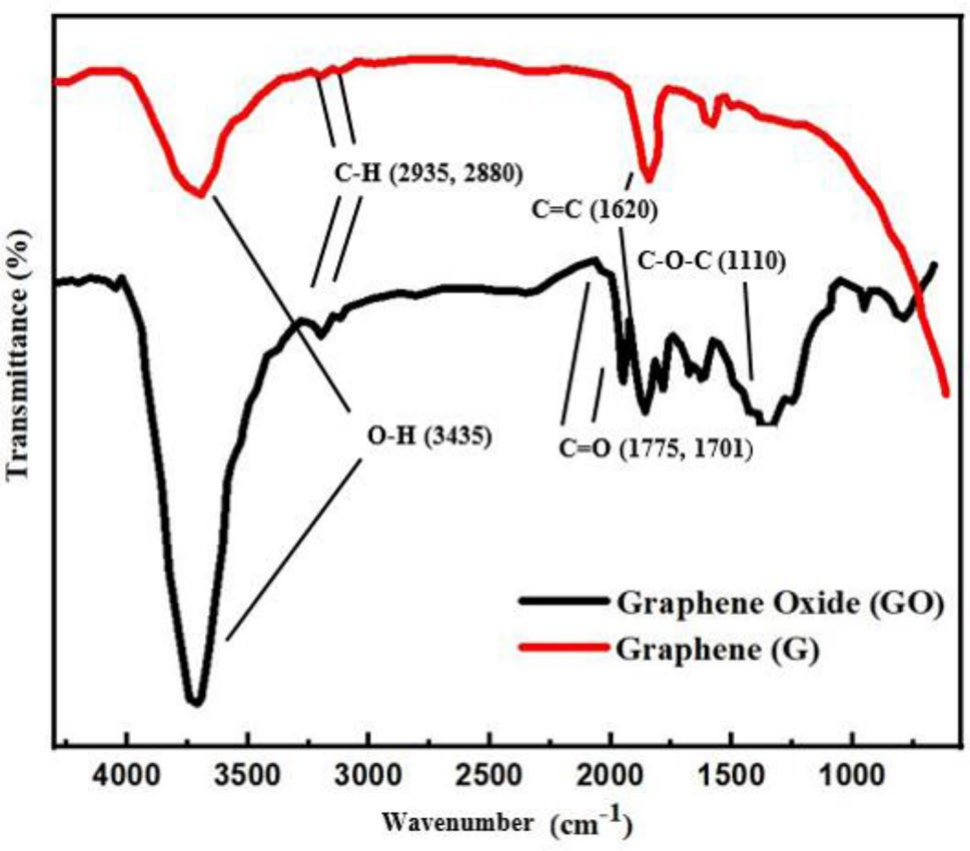
FTIR analysis of graphene and GO.

In order to confirm the functionalization of GO, FTIR analysis of pristine graphene and GO was conducted. FTIR analysis is shown in Fig. 1 in a wavenumber range from 650 to 4000 cm^−1^. In FTIR analysis, a strong peak at 3435 cm^−1^ is assigned to strong hydroxyl group O–H (O–H stretching vibrations)^25^. In pristine graphene same peak was observed but with lower intensity. In contrast, after surface modification, the higher intensity peak shows the degree of oxidation of pristine graphene by oxidation in a highly acidic condition^26^. The peaks at 2935 and 2880 cm^−1^ are attributed to symmetric and asymmetric stretches of C–H and the H–C=C–H group is attributed to the presence of methylene stretches. The two new peaks at 1775 and 1701 cm^−1^ are not observed in pristine graphene, while on graphene oxide indicates carbonyl (C=O) group, which could be related to extending vibrations due to carbonyl group (–COOH)^27^. Whereas, the vibrations at 1775 and 1701 cm^−1^ could be associated with the attachment of C–O groups giving evidence of the presence of oxygen-containing groups at the graphene surface. The peak observed at 1620 cm^−1^ on both pristine and oxidized graphene could be attributed to alkene (C=C), termed as the backbone of the graphene structure. The strong peak at 1110 cm^−1^ also indicates (C–O–C) the presence of strong oxygen-containing functional groups^28^. The surface modification of graphene by a strong acidic mixture of sulphuric and nitric acid has significantly modified the structure of graphene and shows a high degree of covalent functionalization^29^.

### Dispersion stability

A high degree of dispersion and stability of graphene oxide in aqueous suspension is highly desired to obtain an outstanding GO film in the form of buckypaper with good mechanical and electrical properties. In this research, the optimum condition to obtain a well-dispersed solution of graphene was recorded at 60% sonication power, with sonication applied for 15 s after every 30 s for 20 min. The sonication process exploited and disintegrated graphene oxide from aggregates and bundles into freestanding graphene oxide sheets. The low power sonication after one hour clearly showed that graphene oxide has very low dispersibility in methanol and acetone and settles at the bottom of the beaker. Whereas in ethanol, graphene achieved a high degree of uniformity and dispersibility. The dispersibility of oxidized graphene is due to the highly polar nature of ethanol contributed by the presence of carboxylic groups on the graphene oxide surface that creates electrostatic repulsion and polar-polar affinity^30^.

**Figure 2:**
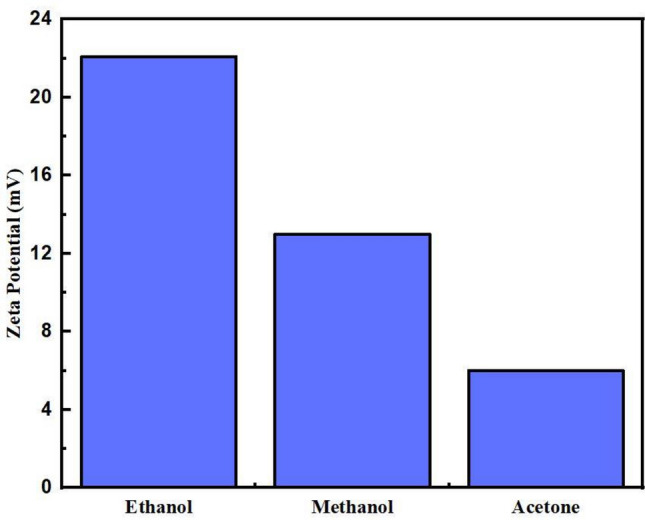
Zeta potential of GO in ethanol, methanol, and acetone.

Figure 2 shows GO dispersion in ethanol, methanol, and acetone with values 22.08, 13 and 6 mV, respectively. The higher the values of zeta potential indicate the degree of repulsion between oxidized graphene nanoparticles in the solvent. As a result, good dispersion of GO without any agglomeration and aggregation was achieved^31^. These results further signify that the electrical double layer consisting of a rigid layer bonded to particles and the electrostatic repulsion produced by the diffused layer, which overcomes van der Waals forces resulting in a homogenous solution of GO. As a concluding remark, relatively charged GO overcomes van der Waals attraction and plays a crucial role in solution stabilization. The zeta potential results lower than − 15 mV and higher than 15 mV are considered acceptable for good quality dispersion. However, a value higher than 40 mV is considered as high-quality dispersion^32^.

### Morphology

**Figure 3:**
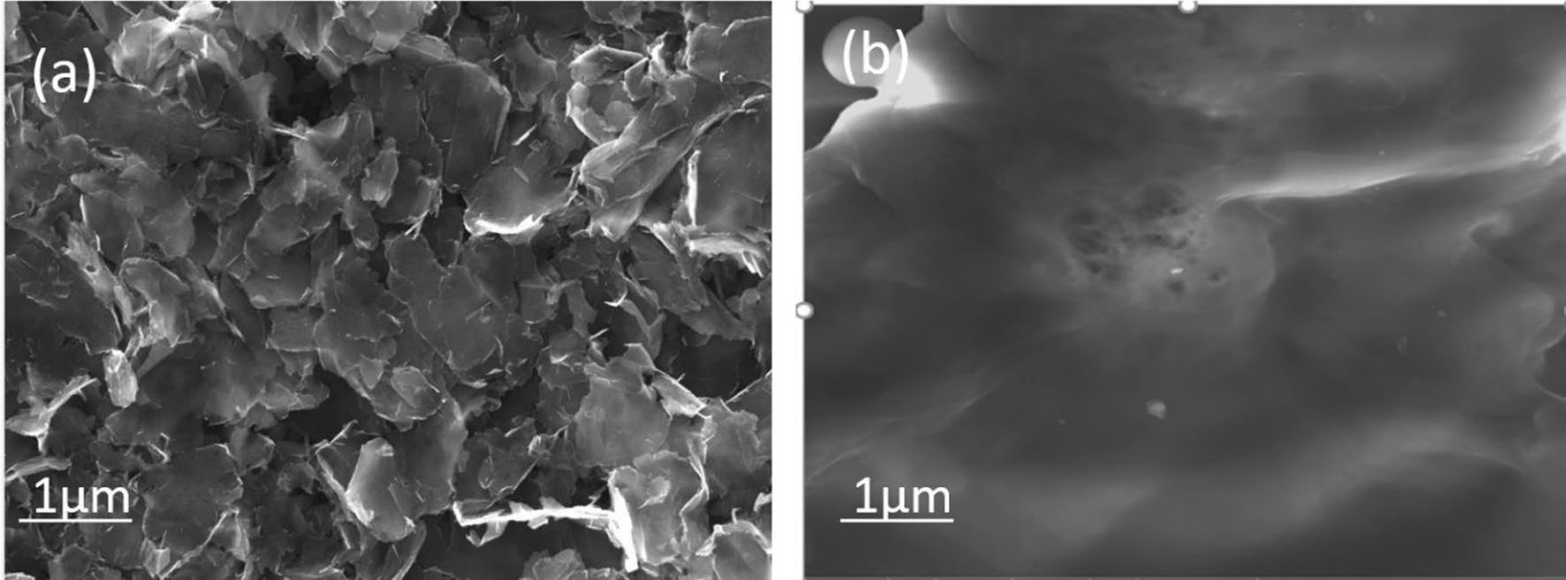
Shows the SEM images of (**a**) 75 BP/PVA and (**b**) 60 BP/PVA.

The SEM image of the surfaces of BP is presented in Fig. 3a,b. Figure 3a shows an SEM image of 75 graphene BP/PVA polymer composite. Graphene sheets can be seen with empty spaces around them and are scattered randomly with good dispersion that makes it possible for obstruction-free current flow^33^. The tunnelling resistance and contact resistance are less in 75 BP/PVA as compared to 60 BP/PVA as shown in Fig. 3b, due to less space between graphene sheets^34^. Thus, the filling of empty spaces forces graphene sheets apart and increases distance among them; therefore, resulting in increased tunnelling and contact resistance.

Figure 3b shows a surface image of 60 BP/PVA, representing no visible pores in the structure of BP, which testifies good dispersion of PVA in graphene sheets, indicating the PVA matrix infiltrated into polymer sheets and filled empty pores in between sheets^35^. Good infiltration of the PVA matrix into graphene sheets ensure good adhesion between BP sheets and PVA matrix and contributes to better mechanical properties.

### Raman analysis

**Figure 4:**
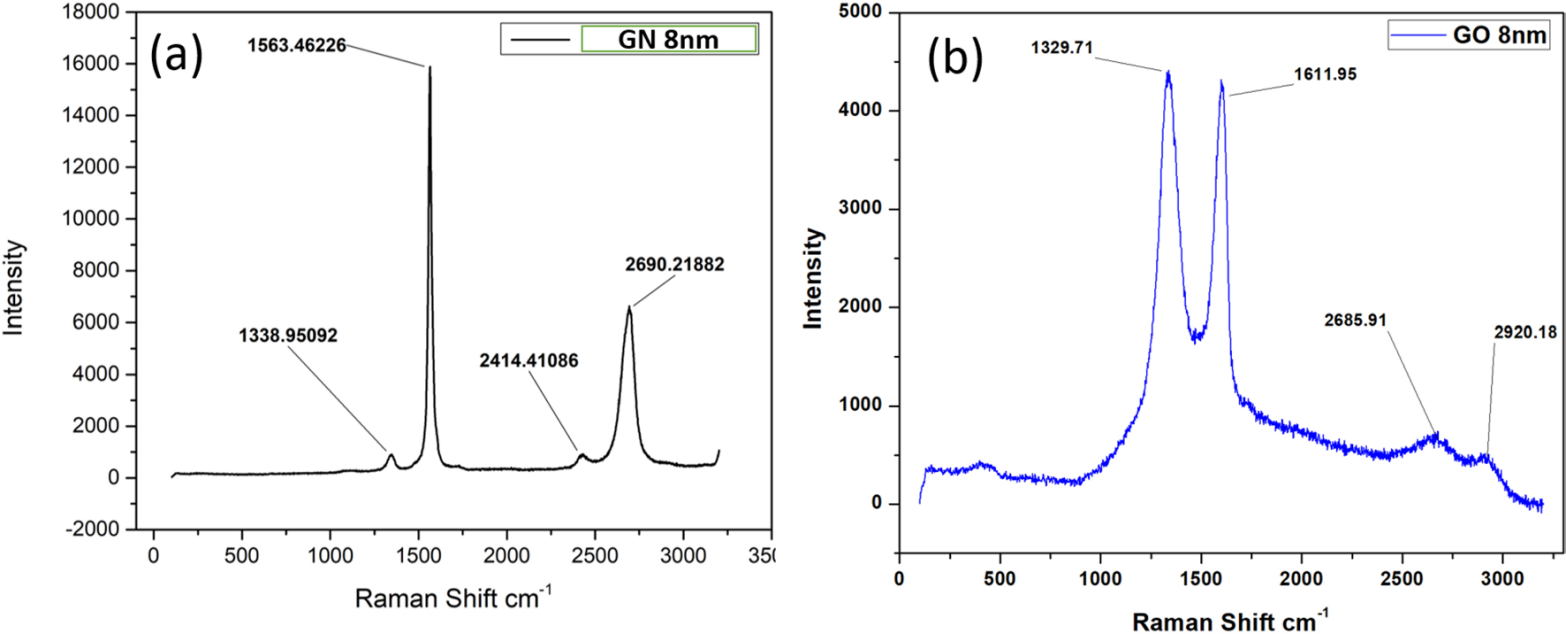
Raman Spectra of (**a**) graphene (GN) and (**b**) graphene oxide (GO).

Figure 4 shows the Raman Spectra of (a) graphene (GN) and (b) graphene oxide (GO). The D and G bands of GN and Acid treated GN in Raman spectra. For GN sample D-band (sp^3^ character) at around 1338.9 cm^−1^ is due to the defects, disorder, impurities etc., present in the samples whiles G-band (sp^2^) at 1563.4 cm^−1^ corresponds to the characteristic peak of most of the carbon related materials due to the in-plane vibration of carbon atoms. G band shifted to a higher energy of 1611.9 cm^−1^ in acid treated GN. This could be due to changes and disturbance in GO sheet. The intensity ratios of D- and G-bands (ID/IG) calculated for pure GN and acid treated GO are 0.05 and 1.01 respectively. The dramatic increase of D-band Raman intensity is interpreted as the presence of defects and disorder formed in the sample after vigorous mechanical and chemical process of acid treatment^36,37^.

## Characterization of PVA infiltrated graphene-based bucky paper

### Elemental composition

Energy-dispersive X-ray analysis (EDX) of GO and PVA infiltrated BP (60 BP/PVA polymer composite) was performed to know the elemental composition. Table 1 shows EDX results of 75 graphene-based BP/PVA, the C-peak between $\sim\; 0.2 \;\text{to} \;0.4 \;\text{keV}$ of high intensity confirms that graphene contains a high mass fraction of carbon atoms 95.19 wt% and O-peak at $\sim\; 0.5\;\text{ keV}$ validates the presence of oxygen atom in a mass fraction of around 4.52 wt%. The presence of oxygen atoms is due to the acidic treatment of graphene with a mixture of hydrochloric acid and sulphuric acid^38^. The low-intensity peak shows the presence of sulphur atoms due to acid treatment for the functionalization of graphene and also sulphur is used as a growth promotor during the synthesis of graphene by a chemical vapor deposition method. EDX composition of 60 BP/PVA polymer composite C-peak $\sim\; 0.2 \;\text{to} \;0.4 \;\text{keV}$ confirms the presence of carbon atoms in a mass fraction less than 75-graphene based BP/PVA around 76.07 wt. %, while O-peak $\sim\; 0.5 \;\text{keV}$ validates the presence of oxygen atoms in higher weight proportion, Oxygen atoms by weight makes around 16.59%, this higher mass fraction could be attributed to first, the oxidation of graphene second to polyvinyl alcohol infiltration, containing (-OH) hydroxyl group^39,40^. The last peak on PVA infiltrated BP at $\sim\; 0.2\; to \;0.4\;\; \text{keV}$ could be attributed to zinc left overused as a metal catalyst and in metal foil in which EDX is carried out.

**Table 1:** EDX of 75 and 60 graphene-based buckypaper.

Elements	Elemental composition weight (%)	
	75-graphene BP/PVA	60-graphene BP/PVA
C	95.19	76.07
O	4.52	16.59
S	0.30	0.23
Zn	–	7.11

### Mechanical properties

**Figure 5:**
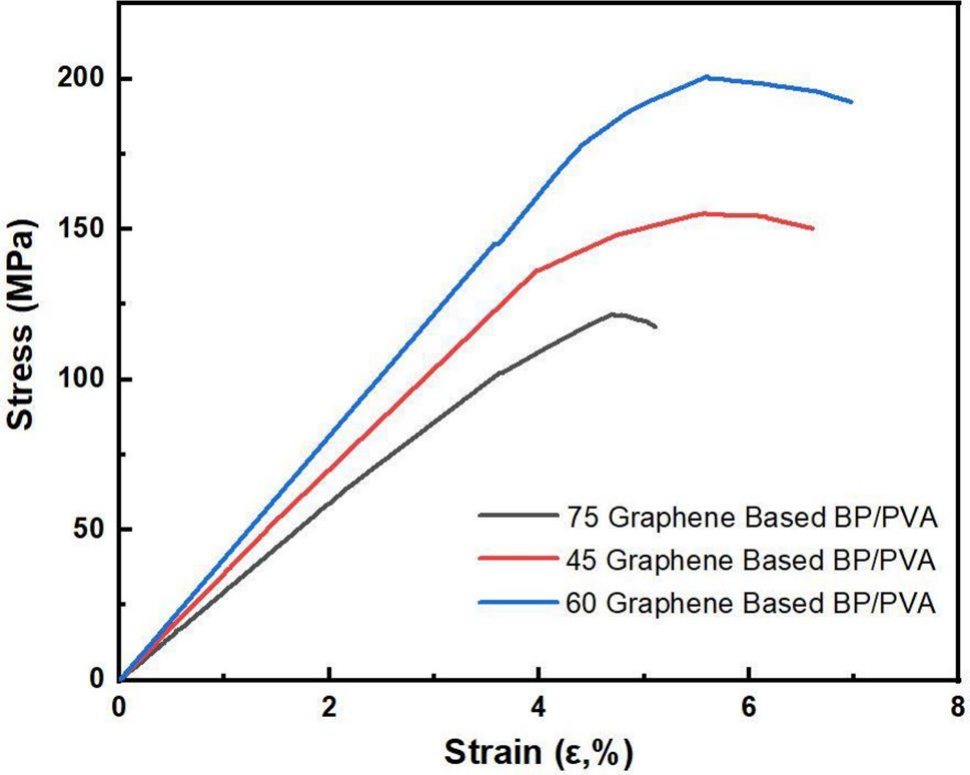
Uniaxial tensile stress–strain of BP/PVA composites.

The tensile testing was performed to determine the performance of various compositions of PVA infiltrated BP, such as 45 wt%, 60 wt% and 75% of BP, respectively. Figure 5 shows the stress–strain curve of various concentrations of graphene-based BP. The Young’s modulus illustrates the stiffness and ability of BP to withstand a permanent deformation under strain^41^. The strain-to-failure ratio represents the maximum elongation ability in contrast to the original length before its complete failure.

The stress–strain curve in Fig. 5 can be divided into two regions, one with elastic deformation and other with inelastic deformation^42^. PVA infiltrated BPs show tensile strengths in a range of 121–200.53 MPa, Young’s modulus within 3.69–6.59 GPa, while all BPs have strain to failure ratio in a range of 4.66–6.79%. The pure 100% BP was not synthesized successfully due to the high degree of slippage between graphene layers, which does not allow layers of graphene to stay together and cannot be peeled off successfully from the substrate. Slippage can be predicted with high certainty as it is an inherent property of carbon nanotubes and graphene materials, even slippage in carbon nanotubes BP is attributed to slippage of graphene layers as graphene is the mother material of carbon nanotubes^43^. The slippage further can be explained as weak van der Waals interaction between layers of graphene^44^. The other possibility of failure to achieve freestanding graphene-based BP without any PVA infiltration is attributed to the agglomeration of graphene layers at a high loading of 100%. The forces of attraction among graphene sheets in 100 wt% BP were so weak that it broke when tried to peel off from the PTFE membrane^45^. Table 2 shows the mechanical properties of PVA infiltrated Graphene-Based BP.

**Table 2:** Mechanical properties of PVA infiltrated graphene-based BP.

Sample name	Tensile strength (MPa)	Young’s modulus (GPa)	Strain to failure ratio (%)
75-BP/PVA	121.38	3.69	4.66
60-BP/PVA	200.55	6.59	6.79
45-BP/PVA	155.27	4.33	5.85

However, PVA infiltrated BPs composites demonstrated linear elastic deformation in the elastic region, and plastic deformation in the non-elastic region, first necking, and finally complete failure^15^. The phenomena of Plastic deformation is permanent in nature, first necking starts to appear in structure and further material shows no resistance to the applied force, and finally breaks apart. These tests help to define the working range of a material in which it can be utilized and exceeding this limit may fail the respective component permanently^29^. All PVA infiltrated BPs demonstrated steeper linear slope with high tensile strength, Young’s modulus, and strain to failure ratio in a range of (ε ~ 4 to 6%). The 75-BP/PVA polymer composite showed a lowest tensile strength of 121.38 MP, Young’s modulus of 3.69 GPa, and strain to failure ratio of 4.66% as shown in Fig. 5, while 45-BP/PVA polymer composite demonstrated tensile strength of 155.27 MPa, Young’s modulus of 4.33 GPa and elongation to break ratio of 5.85%. However, 60 BP/PVA showed steepest linear curve among all and possess tensile strength of 200.55 MPa, Young’s modulus of 6.59 GPa and elongation at break ratio of 6.79%. Further, 60 BP/PVA polymer composite stress–strain curve slope has linear behaviour until the strain of 5.2% than the curve starts to change shape. The gradient of the line started to decrease, indicating a proportional limit of the material has reached; this point gives the value of yield strength of the material. The phenomenon happened at 4% in 75 graphene-based BP/PVA and less than 5% in 45 based BP/PVA. Plastic deformation disconnects physical constituents of material; in the case of PVA/BP, the PVA infiltration that initially provides a connection between layers of graphene starts to disappear until ultimate failure at the weakest point in the structure has occurred. Plastic deformation first starts to form a neck at the weak point that reduces the cross-section of the material until the completely disconnects the of two ends to material^46^.

The superlative mechanical properties can be a result of improved interfacial interaction between Graphene sheets and the PVA matrix. During the infiltration process, the PVA solution diffuses into BP and interacts with GO by binding GO layers together^17^. Additionally, the strong hydrogen bonding between functional groups containing oxygen such as C=O, –OH may also add strength by the contribution of interaction between graphene oxide carbonyl groups and PVA hydroxyl groups^47^. PVA contains a hydroxyl group capable of hydrogen bonding interacts with the functional group present on functionalized graphene such as carbonyl (C=O), hydroxyl (–OH), and carboxylic group. Thus, strong interaction and interfacial adhesion between various functional groups of GO and PVA matrix contribute to good dispersion throughout the BP, resulting in superior mechanical properties.

However, the Graphene-based BP/PVA shows variability in mechanical properties as shown in Fig. 5. PVA infiltration into BP does not follow a monotonic trend in characteristics like Young’s Modulus, strain to failure ratio, and tensile strength. The strain to failure ratio increases with increasing PVA matrix into BP and decreases with increasing graphene content. This is caused by the chain mobility of PVA under the presence of graphene. The higher concentration of PVA provides flexibility to BP/PVA polymer composite structure hence increases the elongation to break ratio of the sample. The sample 60 BP/PVA polymer composite achieved the highest Young’s modulus of 6.59 and tensile strength of 200.55 that can be attributed to the possibility of complete infiltration of PVA into BP and fills all empty pores in the structure and provides continuous interaction throughout the structure by filling empty spaces. The excessive PVA matrix infiltration into BP may create stress in the structure by disturbing the natural harmony. The sufficient amount of PVA polymer improves bonding in the whole structure due to the availability of the PVA matrix at every location in the structure, in the same manner, complete infiltration of PVA is seen in the SEM image of 60 BP/PVA. The mechanical properties of the 60-BP/PVA polymer matrix, such as tensile strength (200.55) are higher than the reported values in literature prepared by mixing method (27.9–51.1 MPa)^48^ while less than prepared by soaking^49^.

Furthermore, Young’s modulus is also higher than the reported in the literature (0.25–1.37) while 60-BP/PVA polymer composite demonstrated Young’s modulus of 6.59 GPa, significantly higher than reported for BP strain sensors. The further addition of PVA into BP in 45 BP/PVA reduced the tensile strength to 155.27 GPa and Young’s modulus to 4.33 GPa. This effect can be attributed to the irregular distribution of the PVA matrix into BP; besides higher concentration of PVA solution blocks the pores of BP at the surface and stops further penetration into BP^50^. Moreover, the solidification of PVA at various locations on the BP surface and the presence of insoluble hydrogels in the PVA solution further reduced or completely blocked the flow of PVA solution into BP. All these factors collectively contributed to incomplete diffusion of the PVA matrix into BP, hence retarding its mechanical properties^51^.

Overall, PVA infiltration into BP phenomenally improved its properties and provided necessary strength to the structure of BP so that it can be peeled off from the PTFE membrane. All the studies in the mechanical section indicate that 60-graphene based BP/PVA polymer composite is a better choice due to its superior properties, with 40% higher tensile strength from 75-graphene-based BP/PVA polymer composite while 23% higher than 45-graphene based BP/PVA polymer composite. In terms of Young’s modulus, it is 31% higher than 75-graphene-based BP/PVA polymer composite while 14% higher than 45-graphene based BP/PVA polymer composite. The better mechanical characteristics of 60-graphene based BP/PVA polymer composite is evident that good dispersion PVA matrix and infiltration into the graphene network and the existence of strong intermolecular interaction between PVA and graphene sheets. The strong interaction between the PVA matrix and graphene makes possible an effective strain signal transfer throughout the structure and makes it feasible for strain sensing application.

### Electrical properties

#### Two-point probe method

**Figure 6:**
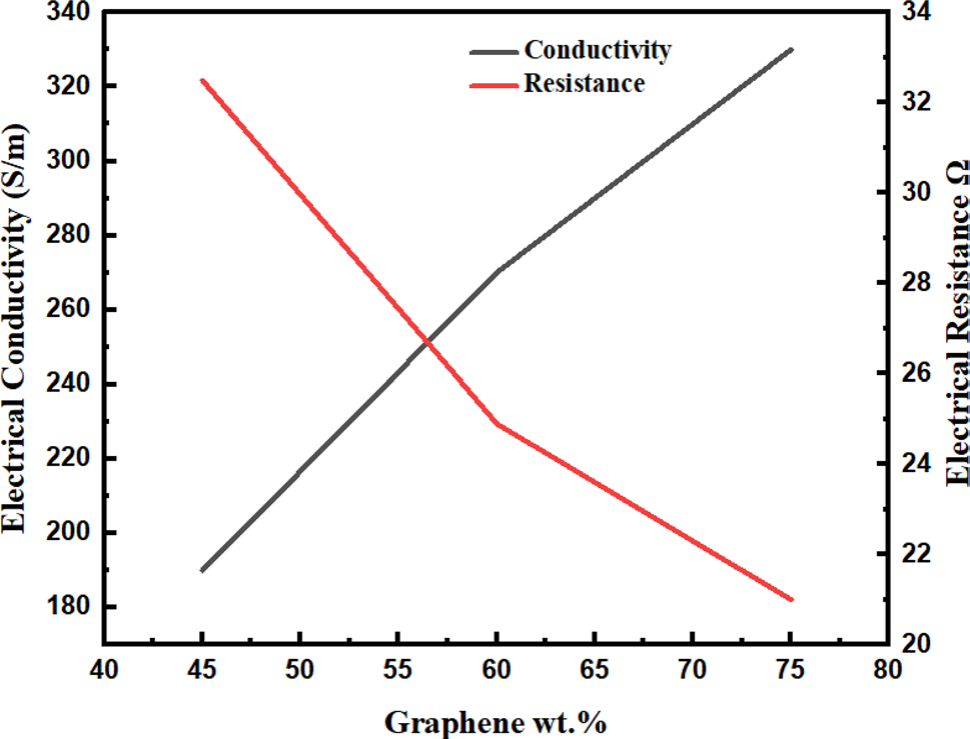
Electrical conductivity and resistance of graphene-based BP/PVA (two-probe point).

BP possesses electrical resistance that is based on three primary elements i.e., contact resistance among graphene sheets, tunnelling resistance among the bordering graphene, and pristine resistance of graphene as an individual^52^. A dense system of conductive graphene in BP offers a smooth flow of current via BP. In order to evaluate BP’s strain sensor electrical efficiency, BP specimen DC electric measurement was conducted through a digital multimeter based on a two-point probe method concept, ASTM D-4496-13. Figure 6 displays sample properties such as conductivity and electrical resistance trends using a two-point probe method. The electrical conductivity shows a direct relationship with BP/PVA concentration as shown in Table 3. It can be explained that the polymer intercalation of graphene-based BP/PVA has increased composite thickness, resistivity, and electrical resistance. Graphene-based BP infiltrated with PVA displayed a decrease in electrical resistance because of the existence of a transparent layer of PVA on the surface of the composite formed in the infiltration process. During the infiltration stage, PVA is attached to the BP surface rapidly and fill the BP pores.

**Table 3:** Electrical properties of graphene-based BP/PVA (2-point probe method).

Sample	Film thickness, mm	Width, mm	Gauge length, mm	Electrical resistance (R), Ω	Bulk resistivity (P_b_), Ωm	Electrical conductivity *σ*, Sm^−1^
75 G-BP/PVA	0.43	5	15	22.75	3.26 × 10^–3^	3.06 × 10^2^
60 G-BP/PVA	0.46	5	15	28.54	4.37 × 10^–3^	2.28 × 10^2^
45 G-BP/PVA	0.49	5	15	57.77	9.43 × 10^–3^	1.05 × 10^2^

The increase in PVA loading, not only increases PVA in BP but also built a thick layer of BP surface, hence they collectively block the current path and increases BP resistance. Furthermore, this is followed by greater bulk resistivity value and lower conductivity. In contrast, uppermost bulk resistivity and lowermost conductivity noticed for 45-graphene based BP/PVA composite reflects a thicker coating of PVA surface deposit^53^.

There was a slight increase in BP/PVA thickness from 0.43 to 0.49 mm, due to more PVA could hold on the nanocomposite surface. Furthermore, a decrease in conductivity in BP/PVA may be attributed to high electrical insulating characteristics of PVA polymer, which is attached at the composite interface hence the bulk and surface resistivity that results in poor electrical conductivity. The electrically insulating PVA penetration into the porous graphene system fills the gaps among adjacent graphene. As a result, electrically conductive paths are decreased; hence, contact and tunnelling resistance increase among adjacent graphene sheets^54^.

#### Four-point probe method

The electrical properties of graphene-based BP specimens were analysed further using a four-point probe method based on Van der Paw Hall effect measurement. The results of electrical studies are illustrated in Table 4.

**Table 4:** Electrical properties of graphene-based BP/PVA (4-point probe method).

Sample	Electron density Ne, (cm^−3^)	Electron mobility *µ*_*e*_, (cm^2^/Vs)	Electrical resistance (R), Ω	Bulk resistivity (P_b_), Ωm	Electrical conductivity *σ*, Sm^−1^
75 G-BP/PVA	7 × 10^21^	2.93 × 10^–1^	21.23	3.04 × 10^–3^	3.06 × 10^2^
60 G-BP/PVA	5.5 × 10^21^	2.97 × 10^–1^	24.94	3.82 × 10^–3^	2.28 × 10^2^
45 G-BP/PVA	6.10 × 10^21^	1.95 × 10^–1^	32.21	5.24 × 10^–3^	1.05 × 10^2^

**Figure 7:**
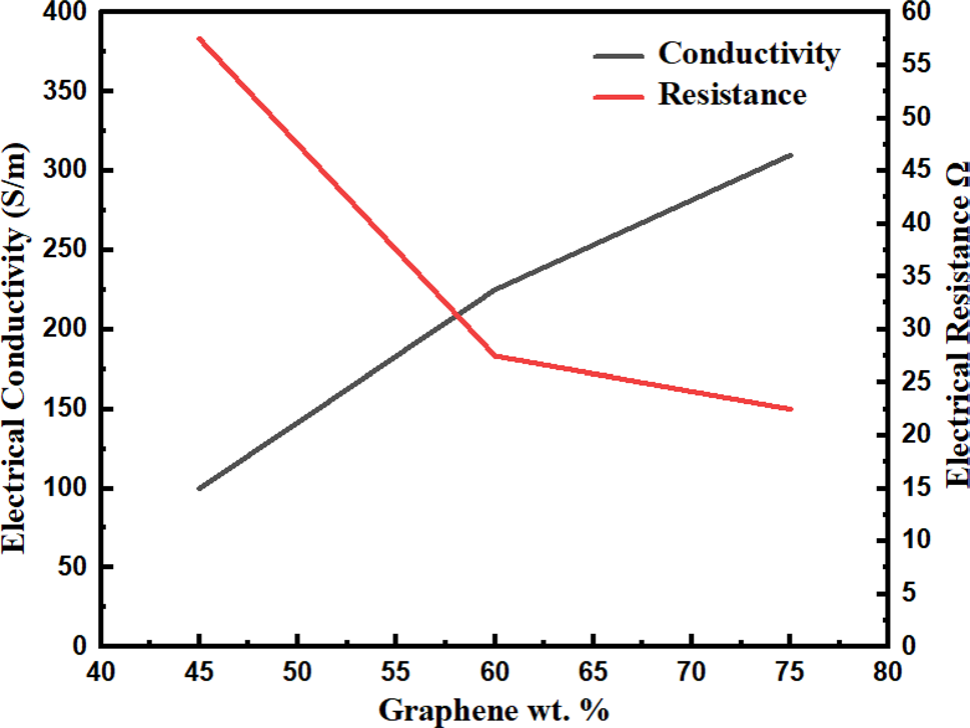
Electrical conductivity and resistance of graphene-based BP/PVA (four-probe point method).

**Figure 8:**
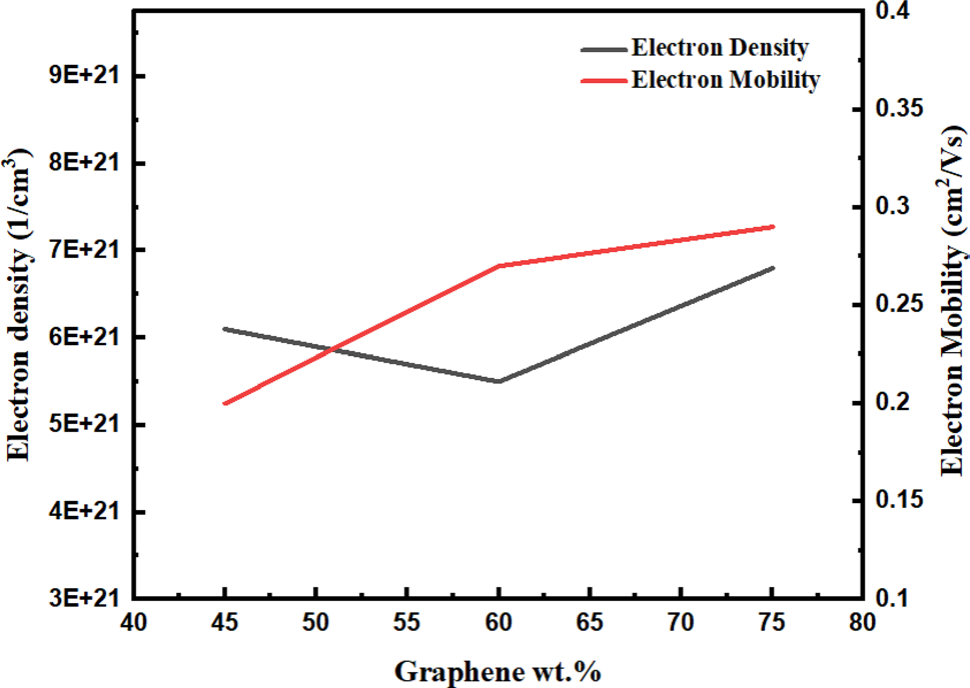
Electrical density and mobility of graphene-based BP/PVA (four-probe point method).

According to Fig. 7, graphene-based BP samples displayed a positive trend regarding electrical conductivity, while showed a negative trend when subjected to an increase in PVA content in BP. Generally, the results are identical to the two-point probe method. Table 4 lists the electrical properties of BP calculated using the four-point probe method^18^. As displayed in Table 4, and Fig. 7, the uppermost conductivity, 328 Ohms found in BP was led to higher electron mobility, 2.93 × 10^–1^ cm^2^/Vs, and density, 7 × 10^21^ cm^−3^ Moreover, greater content of BP forms a denser system of conductivity paths, consequently lower bulk resistivity and higher conductivity. In contrast, PVA infiltration has affected electronic conductivity as depicted in Fig. 8. PVA infiltrated graphene-based BP electrical conductivities are in 10^2^ S/m order, which is appropriate for strain sensing application.

Besides, carrier densities of specimen stayed in 10^20^–10^21^ cm^−3^ range^55^, which are considered comparatively higher than prior studies. Among all graphene-based BP/PVA samples, 60 BP/PVA composite achieved the highest mobility of 2.97 × 10^–1^ cm^2^/Vs but with the lowest carrier density of 5.5 × 10^21^ cm^−3^. This indicates that low-density electron can move more conveniently via the composite with a faster rate, leading to superior strain sensing performance. While BP samples conductivity was obtained to be proportional to electron density product, N_e_, and mobility, *µ*_*e*_, in which q refers to electric charge, as written in Eq. (1).

1 $$\sigma \, = \, \left( {N_{e} } \right)\left( q \right)\left( {\mu_{e} } \right)$$

In brief, both methods have confirmed that the electrical conductivity of BP declined when subjected to a higher volume of PVA solution infiltration. The PVA infiltrated graphene-based BP’s electrical conductivity was controlled through the electrically insulating PVA layer thickness. It is concluded that PVA thicker layer leads to lower conductivity. Hence, PVA layer control requires the optimization of infiltration steps, together with the quantity and concentration of PVA solution, and soaking time. The high degree of polymer intercalation has directed to decline in conductivity of PVA infiltrated BP from $3.06 \times {10}^{2} \;to\; 1.05 \times {10}^{2}\; {\text{Sm}}^{-1}$(2-point probe) and from $3.28 \times {10}^{2} \;to \;1.91 \times {10}^{2}\; {\text{Sm}}^{-1}$. Generally, greater conductivity of BP/ PVA samples was achieved via a 4-point probe, which may be ascribed to the removal of contact resistances from the measurement. 75 BP/ PVA and 60 BP/PVA obtained higher conductivity than the recorded BP/TPU (Thermoplastic polyurethane) nanocomposite via the same soaking procedure^56^. Taking the material and preparation methods into consideration, the values of conductivity obtained are within experimental accuracy.

### Electromechanical properties

Electromechanical behaviour is one that testifies the strain sensor application of a material. The piezoresistive behaviour of the sensor is tested to validate its performance as a strain sensor. In this test, the strain is applied on a sensing material with both ends connected to resistance measuring instrument and change in resistance of a material is recorded. Different conducting materials have a different response to applied strain, the material with quick, precise, accurate, repeatable, and sensitive response to applied strain is one with good characteristics of a sensor. In this study, all three compositions demonstrated strong piezoresistive behavior and showed promising results for their application as a strain sensor^57^. The resistance of all samples changed linearly with an increase in applied strain. The change in resistance of BP/PVA polymer composites can be attributed to an increasing distance between layers of graphene and a decrease in the density of the graphene conductive network. The applied stress decreases the cross-sectional area and increases the length of the sample. The continual increase in stress widens gaps between graphene sheets, resulting in a decrease in conductive paths. Consequently, the reduction in conductive path reduces current flow through BP which in turn increases the electrical resistance of the graphene-based BP/PVA.

Further to measure the sensitivity of graphene-based BP/PVA, the gauge factor based on relative resistance change and strain is performed in a strain range of (ε ~ 4%), as 75-graphene-based BP/PVA starts to deform its shape after (ε ~ 4%) and linear relationship between strain and relative resistance change no longer holds^58^. The gauge factor itself is a slope of relative resistance change curve versus strain. The following table shows the gauge factor of various strain sensors.

Table 5 above shows the gauge factor of various samples, 75 BP/PVA as 0.16, 60 BP/PVA as 1.33, and 45 BP/PVA as 2.51. The sample 75 BP/PVA demonstrates the lowest gauge factor of 0.16 among all samples. The increase in resistance under tension can be associated with tunneling resistance developed due to the increase in distance between layers of graphene and the increment is reported exponentially with increasing distance between particles. Under mechanical strain, the increase in tunneling resistance can be associated with an increase in gaps between graphene layers that were earlier filled by the PVA matrix. However, PVA itself is electrically insulating and the greater the amount of PVA matrix, the higher the gauge factor obtained^59^. In addition to tunnelling resistance, the higher value of the gauge factor can be attributed to increased contact resistance among layers of graphene, additionally, the PVA matrix fills vacant spaces in BP. In stretch position, graphene layers become completely isolated with no conductive paths and even worse happens when graphene becomes physically apart.

**Table 5:** Gauge factor of PVA infiltrated graphene-based BP.

Sample name	Gauge factor
75-Graphene BP/PVA	0.16 ± 0.05
60-Graphene BP/PVA	1.33 ± 0.05
45-Graphene BP/PVA	2.51 ± 0.05

**Figure 9:**
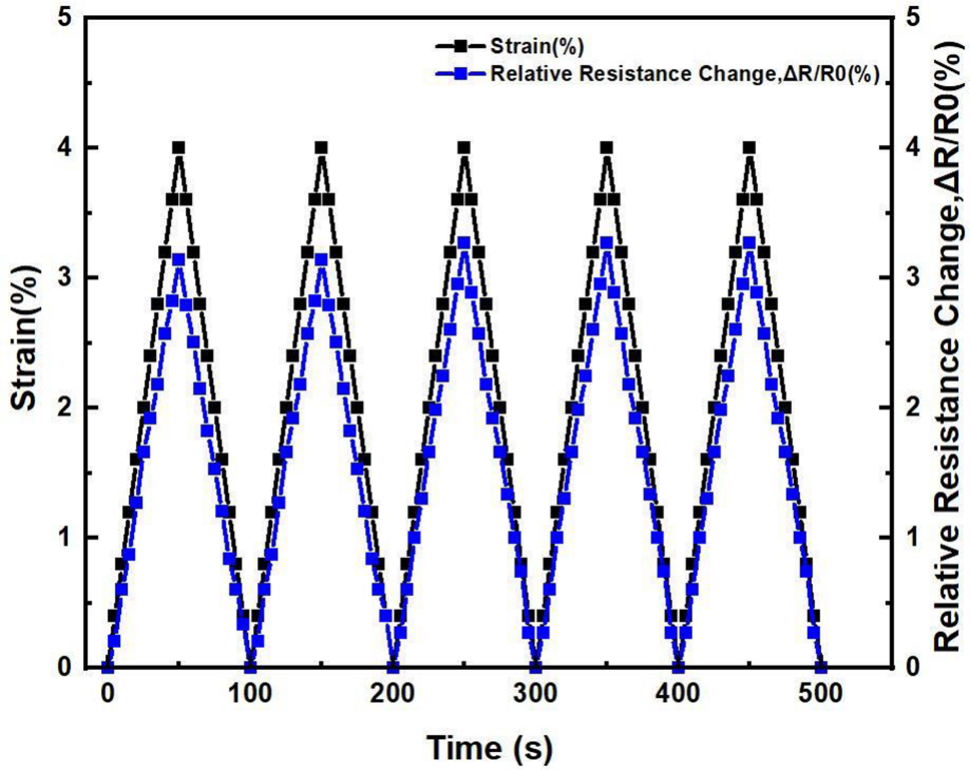
Piezoresistive response of 60 graphene BP/PVA.

**Figure 10:**
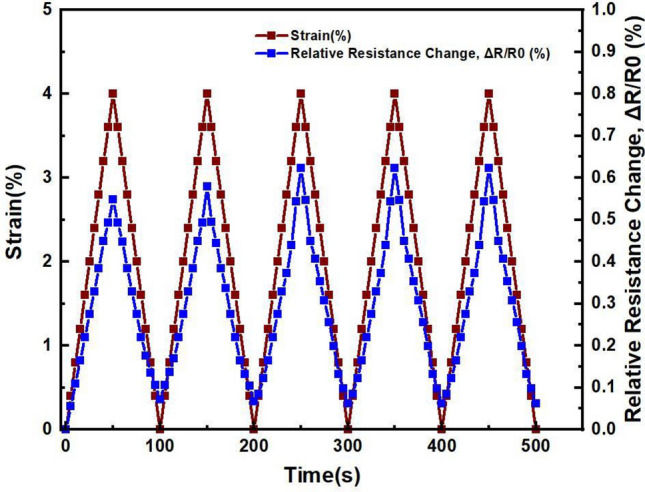
Piezoresistive response of 75 graphene-based BP/PVA.

Furthermore, to fully reveal electrochemical characteristics of BP/PVA polymer composite for strain sensor application. Cyclic uniaxial tensile testing was performed to demonstrate and investigate the piezoresistive behaviour of graphene-based BP/PVA polymer matrix during multiple unloading and loading cycles^60^. The loading and unloading cycles were demonstrated in the strain range of (ε ~ 4%) which is within the elastic region. The relationship between tensile strain and relative resistance change vs time of 60-graphene-based BP/PVA polymer composite is shown in Fig. 9 and for 75-graphene-based BP/PVA is shown in Fig. 10.

Figure 9 shows that the strain is applied in a range of ε 4.0%, in a cyclic order of loading and unloading, for a period of 500 s, one cycle of loading and unloading consists of 100 s and five similar cycles were applied on each sample. A linear slope of increasing and decreasing relative resistance change was observed as a whole, however with the application of loading and unloading strain in a range of 4% and relative resistance change of 3.5% was observed. Similarly, 60-graphene-based BP meets another criterion of the sensor, namely repeatability and along with it also satisfies accuracy by following the same path for each cycle. Additionally, a similar shape cycle pattern for all five cycles was observed without any relative resistance change drifting. Whereas, the small phase difference is observed between applied strain and the piezoresistive response of strain sensor composite. In this testing, no hysteresis phenomena are observed, also, no hysteresis effect during loading and unloading cycles in an elastic range of $\varepsilon \sim 4\%$ is observed for all five cycles, further validates the strain sensor application of 60-graphene-based BP/PVA. The repeatability in piezoresistive response and linear relative resistance change under five loading and unloading cycles validates the high performance of 60 BP/PVA for strain sensor application.

However, on the other hand, 75 BP/PVA did not give satisfactory results. Initially, after the first cycle strain did not return to zero and stopped at 0.1%, indicating the deformation in the shape of the sample. A slight incremental change is also observed in relative resistance change after each cycle^61^, suggesting two possible phenomena that might be responsible, the first is the hysteresis that the sample does not return to its original value after each cycle and the second reason can be attributed to internal structure destruction of PVA infiltrated BP that results in permanent deformation of the sample. The cyclic loading and unloading of the sample increased its original length that increased the gap between layers of graphene, which consequently increased contact and tunnelling resistance, thus higher relative resistance change is observed in the second, third, fourth, and fifth cycle^62^. In the end, it can be concluded that 60-graphene-based BP/PVA demonstrated better characteristics of a strain sensor, showed reproducibility and hysteresis free operation.

In general, the sensing ability of PVA infiltrated BP, mainly depends on three aspects, increase or decrease in contact resistance between graphene sheets, piezo resistivity of individual graphene sheets under strained conditions, and change in tunnelling resistance between graphene sheets due to increase in the gap^63^. Overall, all PVA infiltrated graphene-based BPs demonstrated a good piezoresistive response in the strain range of (ε ~ 4%), but 60 graphene-based BP/PVA demonstrated the highest test results under five loading and unloading cycles and showed recovery in characteristics after each loading and unloading cycle^64^. All these parameters validate the applicability of graphene-based BP infiltrated with PVA for strain sensing applications.

## Conclusion

In summary, a high-quality flexible, and stretchable strain sensor was fabricated by vacuum filtration technique and PVA intercalation. 60 percent concentration of BP with infiltration of PVA showed the best strain sensing characteristics. The sensor demonstrated mechanical strength, Young’s and elongation at break of 200.55 MPa, 6.59 GPa, and 6.79%, respectively, in comparison to buckypaper based strain sensors found in the literature. The sensor showed electric conductance characteristics due to the conducting properties of graphene, while conductivity decreased with increasing PVA content. The 60 BP/PVA infiltration demonstrated a gauge factor of 1.33 and the piezoresistive response was repeatable with no hysteresis for 5 cycles. These characteristics of 60 BP/PVA polymer composite proves to be the best choice among others with improved strain transfer, linearity, repeatability, highly reproducible results, and recoverable structure within strain range. It has commercial applicability to measure critical human health parameters and predict symptoms of chronic diseases, structural health monitoring, wearable technology, and industrial pressure sensors.

## Methodology

### Preparation of functionalized graphene

A solution of sulphuric acid (98%) and Nitric acid (70%) in a proportion of 3:1 (v/v) was prepared and graphene was immersed in it. Immersion resulted in an exothermic reaction and introduced oxygenated groups on the graphene structure through covalent functionalization. The solution with graphene was bath sonicated for 3 h at room temperature. The combination of acid treatment, along with bath sonication helps to create bond cleavage in carbon lattice via electrophilic attack. It also provides an opportunity to carbonyl group for attachment and at the same time, removes residual metallic catalysts. The solution was poured into large beakers with deionized water to dilute the solution and bring down pH near to neutral. The solution was left overnight to let the graphene oxide settle down. The supernatant was discarded, and the precipitate was collected and washed with copious amount of the deionized water. The process of washing with deionized water was repeated until the pH above 5 was achieved. Finally, the solution was poured through the PTFE filter membrane and the graphene oxide was put into a Freezer at − 80 ºC for 24 h, followed by freeze-drying for 24 h to remove the remaining water content.

### Preparation of graphene oxide bucky paper

Three samples of oxidized graphene, each weighing 100 mg were immersed in three 50 ml solvents, namely acetone, methanol, and ethanol. All three samples were sonicated in low power ultrasonic bath cleaner for an hour. The ethanol sample was found to be one with better dispersion of graphene oxide visually. All three samples were further sonicated by high power probe sonication (500 W, 20 kHz) for 15 s with a 30-s break for a total duration of 20 min at an ultrasonic amplitude of 60% (Sono Mechanics, LSP-500). After zeta potential analysis, ethanol was found to one with the best solubility and dispersion. Later the solution of graphene oxide with ethanol was filtered through Polytetrafluoroethylene (PTFE) membrane filtration using a vacuum pump (ULVAC-41B).

The choice of polymer is critical as it plays a major role in determining the BP properties. PVA is a biodegradable, low-cost synthetic polymer with a chemical formula of (C_2_H_4_O)n. PVA is used widely in textile, coating, and papermaking. In BP synthesis, it serves the purpose by developing strong interfacial interaction between graphene and polymer matrix due to functional groups. A 2 wt% solution of PVA in water was prepared such that the solution is not too viscous and there is no residue left. The solution, according to the required weight for infiltration was poured on to the BP formed on the PTFE membrane and was allowed to infiltrate into BP. Samples with different weight fractions of PVA were dried in a vacuum oven at 40 °C for 24 h. The weight fraction of graphene in infiltrated samples was found by subtracting the amount of graphene used initially to cast a BP.

## Materials

Graphene was purchased commercially from the graphene supermarket (Diameter 8 nm, purity 99%). Analytical grade concentrated sulphuric acid ${\text{H}}_{2} {\text{SO}}_{4}$ (98%) and concentrated nitric acid $\text{HNO}_{3}$ (70%) were purchased from Fisher Scientific. Polyvinyl alcohol (99% hydrolysed) with molecular weight (average) 85,000–124,000 g/mol was received from Sigma Aldrich. Standard sodium hydroxide (EMSURE ISO) pellets were purchased from Merck Millipore. PTFE filter membranes with an average pore size of 0.45 µm were received from Merck Millipore. Methanol (99.8%), acetone (99.4%), and ethanol (99.5%) were obtained from Merck Sdn Bhd Malaysia.

## Methods

The Fourier transform infrared analysis was carried out by using FTIR spectroscopy (PerkinElmer) to find out functional groups attached to graphene and graphene oxide. The IR spectra were obtained between wavenumber 650 and 4000 cm^−1^. Further, the zeta potential of dispersed graphene oxide in various solvents was collected using Zetasizer Nano ZS (ZEN3600 Malvern) to identify the solvent with the highest solubility and dispersibility. Whereas the morphology of the BP was observed using a scanning electron microscope (FEI Quanta 400 SEM). EDX analysis was done using EDX spectroscopy (Oxford-Instruments INCA 400 equipped with X-Max Detector). The graphene and graphene oxide samples was determined by using Raman spectroscopy model Renishaw inVia, USA at a wavelength of 5142 nm with green laser excitation. While for mechanical characterization, the strips of rectangular-shaped BP (20 mm × 5 mm) were prepared following standard ASTM D-882 The samples were analysed for tensile loading using a computerized tensile testing machine (Lloyd LR10K Plus). A constant tensile load at a rate of 0.1 mm/min was applied until the sample fractured. Further, for electrical characterization, two aluminum foil strips were attached to opposite ends of samples using silver adhesive to reduce contact resistance and reinforce the connection binding. Direct current electrical resistance was measured using a digital multimeter (Sanwa Japan CD800a), following two probe method according to the ASTM D4496-13 standard. The bulk resistivity (${\rho }_{b})$ and surface resistivity ($\rho s$ ) was calculated using Eqs. (2) and (3).

2 $${\rho }_{b}=\frac{R\times W\times t}{L}$$

3 $$\rho s=\frac{R\times W}{L}$$

The thickness of BP was measured by using a digital micrometer (Mitutoyo Japan). Whereas, electrical conductivity was measured using Eq. (4).

4 $$\sigma =\frac{1}{{\rho }_{b}}$$

Further, electrical properties (resistivity, electron density, conductivity, and electron density) were measured by the Four probe technique using the van der Pauw Hall measurement system (Ecopia HMS 3000 Korea), at a temperature of 300 K under magnetic field. The source of direct current between 1 to 15 mA was passed through outer probes and the resultant voltage drop was measured, however, Ohm’s law ($R=\Delta V/I)$ could be used to measure the resistance of the samples. However, for electrochemical characterization, a gauge factor (GF) is used to quantify the sensitivity of the strain sensor, which is expressed as the ratio of relative change in electrical resistance to an applied strain. The relation is expressed using Eq. (5).

5 $$GF=\frac{\Delta R/{R}_{o}}{\varepsilon }$$

The electrochemical characterization was measured in the form of resistance change with the help of a digital multimeter (Agilent 34401 A) under applied strain. The gauge factor was measured in the linear elastic range of ($\varepsilon \approx 4\%).$ The uniaxial tensile load was applied for five loading and unloading cycles in an elastic range of ($\varepsilon \approx 4\%)$ for a period of 500 s. Each characterization was performed five times on the samples to ensure reproducibility and data consistency.
